# Ordinal logistic regression model describing factors associated with extent of nodal involvement in oral cancer patients and its prospective validation

**DOI:** 10.1186/s12874-020-00985-1

**Published:** 2020-04-26

**Authors:** Vishwajeet Singh, Sada Nand Dwivedi, S. V. S. Deo

**Affiliations:** 1 Department of Biostatistics, All India Institute of Medical Sciences, New Delhi, 110029 India; 2 Department of Surgical Oncology, Dr BRA-IRCH, All India Institute of Medical Sciences, New Delhi, India

**Keywords:** Ordinal logistic regression, Oral cancer, Nodal involvement, Squamous cell carcinoma

## Abstract

**Background:**

Oral cancer is the most common cancer among Indian men, and has strong tendency of metastatic spread to neck lymph node which strongly influences prognosis especially 5 year survival-rate and also guides the related managements more effectively. Therefore, a reliable and accurate means of preoperative evaluation of extent of nodal involvement becomes crucial. However, earlier researchers have preferred to address mainly its dichotomous form (involved/not-involved) instead of ordinal form while dealing with epidemiology of nodal involvement. As a matter of fact, consideration of ordinal form appropriately may increase not only the efficiency of the developed model but also accuracy in the results and related implications. Hence, to develop a model describing factors associated with ordinal form of nodal involvement was major focus of this study.

**Methods:**

The data for model building were taken from the Department of Surgical Oncology, Dr.BRA-IRCH, AIIMS, New Delhi, India. All the OSCC patients (duly operated including neck dissection) and confirmed histopathologically from 1995 to 2013 were included. Further, another data of 204 patients collected prospectively from 2014 to 2015 was considered for the validation of the developed model. To assess the factors associated with extent of nodal involvement, as a first attempt in the field of OSCC, stepwise multivariable regression procedure was used and results are presented as odds-ratio and corresponding 95% confidence interval (CI). For appropriate accounting of ordinal form, the ordinal models were assessed and compared. Also, performance of the developed model was validated on a prospectively collected another data.

**Results:**

Under multivariable proportional odds model, pain at the time of presentation, sub mucous fibrosis, palpable neck node, oral site and degree of differentiation were found to be significantly associated factors with extent of nodal involvement. In addition, tumor size also emerged to be significant under partial-proportional odds model.

**Conclusions:**

The analytical results under the present study reveal that in case of ordinal form of the outcome, appropriate ordinal regression may be a preferred choice. Present data suggest that, pain, sub mucous fibrosis, palpable neck node, oral site, degree of differentiation and tumor size are the most probable associated factors with extent of nodal involvement.

## Background

In India, in terms of prevalence and incidence, oral cancer ranks first among men and overall at rank three, however regarding related mortality it is at third position in men (GLOBOCAN 2012) [1]. The prognosis of oral cancer including 5 year survival rate may get highly affected due to its metastatic spread to neck lymph node [2]. As a result, the regional control and overall survival may also get affected [3–7]. In other words, the outcome of oral cancer may significantly change due to the nodal involvement. Hence, appropriate management of the cervical lymph nodes is an important part of oral cancer therapy [8–13]. However, there has been controversy over the indication, timing and methods of neck dissection [12, 13]. Difficulties in early diagnosing thus restrict the treatment carried out by surgeons [14]. A reliable and accurate means of preoperative evaluation of cervical lymph node metastasis is therefore crucial for the correct management of oral cancer [8, 10, 13, 15, 16]. Also, understanding of its associated factors may provide clues to the clinicians for better management.

There are negligible numbers of studies dealing with analysis of nodal involvement among oral cancer patients. Most of them have focused either on occult nodal metastasis only or a specific oral site i.e., tongue, lip or buccal mucosa or stages [2, 17–27]. Further, they have dealt with only presence/absence of nodal involvement, not in the ordinal form. It is well known that, ignoring the ordering has its own disadvantage mainly because it does not fully utilize the available information [28]. For example, Armstrong and Sloan [29] have reported that compared to a cumulative logit model for a five level ordered response, use of logistic model resulted into a loss of 25–50% of efficiency. To the best of our knowledge, this is the first such study dealing with the ordinal form of extent of involved nodes among oral cancer patients.

Keeping in view of the above points, objective of this study was to develop an ordinal logistic regression model to find out the factors associated with ordinal form of involved nodes and validate it on temporal data.

## Methods

### Data

The utilized dataset under the present study is same as that considered while assessing the factors associated with nodal involvement (yes/no) among oral squamous cell carcinoma patients [30, 31]. As such, the data maintained at Department of Surgical Oncology, Institute Rotary Cancer Hospital (IRCH), AIIMS, New Delhi, India for patients with histopathologically proven oral squamous cell carcinoma who went under surgery including neck dissection was considered. Out of data of 1123 oral cancer patients available during 1995 to 2013, 945 fulfilled the inclusion criteria. Further, prospectively collected data of 204 patients from January 2014 to December 2015 was used for the temporal validation of the developed ordinal model. The number of involved nodes of each patient was collected from their histopathological reports. In view to utilize maximum available information for more accurate results, ordinal categories mainly relied on desired varying management strategies being adopted by the oncologists according to cut-off values of involved nodes. Accordingly it was considered as 0, 1, 2–4 and > 4 involved nodes in ordinal categories. The covariates in this study remain same as those under dichotomous model [30, 31]. Statistical software, STATA/SE version 14.2 (StataCorp LP, College Station, TX, USA), was used for the analysis.

### Statistical analysis

Categorical variables were described using absolute/relative frequency distribution and quantitative variables using measures of central tendency/location like mean (standard deviation)/median (quartile range). The association between qualitative independent variables, was assessed using Chi-square test/ Fisher’s exact test. To assess association between two quantitative variables, Pearson/Spearman’s correlation coefficient was used. Collinearity between the covariates was assessed by Cramer’s V as 0.7 and more. To find out the factors associated with ordinal form of nodal involvement, stepwise ordinal logistic regression procedure was used. Variables which were found to be significant at the level of 25% under crude association analysis (univariable analysis) and/or on the basis of their clinical relevance were taken as a sub-set of covariates for stepwise regression. Results are presented in the form of odds ratio and corresponding 95% confidence interval (CI). Brant test was used for proportionality assumption [32]**.** The model performance was assessed using measure of discrimination and calibration (i.e. the accuracy of the prediction probability of nodal involvement). Discrimination performance was evaluated using Average Dichotomous C-index, Generalized C-index and Set wise C-index (ORC) [33]. Calibration of the predicted probabilities under the developed model was investigated using M.W. Fagerland and D.W. Hosmer test for goodness of fit [34] and specification error by linktest [35]. The equal spaced integer weight score was used to discriminate the individuals regarding calibration and discrimination ability of the developed model [36].

### Statistical models

There are different ordinal logistic regression models to tack care of ordinal form of outcomes. These different ordinal regression models have different ways to form their logits. For example, proportional odds model (considered as cumulative higher category(s) verses cumulative remaining lower category(s)); continuation ratio model (considered as cumulative higher category(s) verses just lower category alone); and adjacent category model (between any of two consecutive categories). Accordingly every form of the logit has its own benefits or limitation, one can use the models as per specific need. To be more specific, continuation ratio model and adjacent category model do not relay on complete data set. In epidemiological and biomedical applications, proportional odd model (POM) is often used. However, sometime continuation ratio model is also used [37, 38]. Further, choice between the POM and CRM models relies on the goals of the statistical analysis. As obvious, in case of nodal involvement, interpretation under POM will remain more logical and meaningful. However, if the proportionality assumption does not fulfill, partial proportional odds model may be a better choice [35, 39]. Further, choice between proportional odds and partial proportional odds models was assessed using likelihood ratio test, LR, AIC and BIC.

#### Proportional odds model (POM)

If log odds ratio across the cut points is identical, i.e. proportional odds assumption is satisfied, the proportional odds model is used. The proportional odds model was initially proposed by Walker and Duncan [40] as cumulative logit model, but later it was named as proportional odds model by McCullagh [37]. In case of the present study, independent observations on 945 patients were available. As described earlier, observation on the nodal involvement (Y) for each patient is classified into either of four categories. Likewise, covariates (*x*_*i*_) denote the p-dimensional vector of covariates (i = 1,2….p), containing the observation on the full set of p explanatory variables. Accordingly, the dependency of Y on *x*_*i*_ may be expressed as:

$$ \Pr \left(Y\ge {y}_j|{x}_i\right)=\frac{1}{1+\mathit{\exp}\left(-{\alpha}_j-{x_i}^{\prime}\beta \right)},j=1,2,3 $$

Or

$$ \log \left[\frac{\mathit{\Pr}\left(Y\ge {y}_j|x\right)}{1-\mathit{\Pr}\left(Y\ge {y}_j|x\right)}\right]={\alpha}_j+{x_i}^{\prime}\beta, j=1,2,3 $$

Where, *Pr*(*Y* ≥ *y*_*j*_) is the cumulative probability of the event (*Y* ≥ *y*_*j*_); *α*_*j*_ are the respective intercept parameters; *β* is a (p by 1) vector of regression coefficients corresponding to *x*_*i*_ covariates.

#### Partial proportional odds model (PPOM)

If identical log odds ratio assumption under POM is not fulfilled for some of the covariates, partial proportional odds model may be used [41]. Out of the two approaches available in this regard, unconstrained partial proportional odds model and constrained partial proportional odds model, unconstrained PPOM model was used due to unavailability of prior knowledge or beliefs regarding constraints and also availability of computational facility [42]. The PPOM permits non proportional odds for a subset of q of the p-predictors (q ≤ p). The unconstrained PPOM cumulative probability may be defined as:

$$ \Pr \left(Y\ge {y}_j|{x}_i\right)=\frac{1}{1+\mathit{\exp}\left(-{\alpha}_j-{x_i}^{\prime}\beta -{t}^{\prime }{\gamma}_j\right)},j=1,2,3 $$

Where *x*_*i*_ is a (p by 1) vector containing the values of observation *i* on the full set of p explanatory variables, *β* is a (p by 1) vector of regression coefficients associated with p variables. Further, *t*^′^ is a (1 by q) vector of q-covariates, containing the values of observation *i* on that subset of the p explanatory variables for which proportionality assumption is either not fulfilled or is to be tested; and *γ*_*j*_ is a (q by 1) vector of regression coefficients associated with the q covariates. Accordingly, *t*^′^*γ*_*j*_ is the increment associated with jth cumulative logit (1 < = j < =3),where *γ*_1_ is equal to 0. If *γ*_*j*_ = 0 for all j, then this model reduces to proportional odds model. Accordingly, the proportional odds assumption for the q variables in t is a test of the null hypothesis that *γ*_*j*_ = 0 for all j = 2, 3.

## Results

The ordinal logistic regression model was developed using data on 945 patients, where as the data of another 204 patients was used for temporal validation of the developed model. Out of 945 patients, females were 212 (22.4%). Majority of the patients were in the age group 40 to 60 years 549 (58.1%) and in lower/lower middle socio-economic class 751 (79.5%). The distribution of number of involved nodes were as; no node involved: 569 (60.21%); 1 node involved: 149 (15.77%); 2–4 nodes involved: 162 (17.14%) and > 4 nodes involved: 65 (6.88%).

Out of 39 available variables, a set of 19 covariates were considered for stepwise regression procedure, among which 9 variables were selected on the basis of its crude association at 25% level of significance and 10 variables on its clinical relevance. Collinearity and first order effect modifier were assessed before developing the multivariable model. They were absent in the present dataset. The distribution of nodal involvement and its associations with covariates are presented in Table 1.

**Table 1:** Distribution and association of nodal involvement with various covariates

Variables		Freq (%)	No Nodes	1 Node(+)	2–4 Node(+)	> 4 Node(+)	*P* value
Gender	Female	212 (22.4)	116 (54.7)	31 (14.6)	44 (20.8)	21 (9.9)	
	Male	733 (77.6)	453 (61.8)	118 (16.1)	118 (16.1)	44 (6.0)	0.062
Age	< 40	184 (19.5)	113 (61.4)	31 (16.8)	29 (15.8)	11 (6.0)	
(years)	40–60	549 (58.1)	322 (58.6)	90 (16.4)	94 (17.1)	43 (7.8)	
	> 60	212 (22.4)	134 (63.2)	28 (13.2)	39 (18.4)	11 (5.2)	0.682
Soc-Eco	LC	252 (26.7)	151 (59.9)	37 (14.7)	45 (17.9)	19 (7.5)	
Status	LMC	499 (52.8)	309 (61.9)	70 (14.0)	84 (16.8)	36 (7.2)	
	UMC	174 (18.4)	97 (55.7)	38 (21.8)	31 (17.8)	8 (4.6)	
	UC	20 (2.1)	12 (60.0)	4 (20.0)	2 (10.0)	2 (10.0)	0.451
Pain	No	521 (55.1)	330 (63.3)	80 (15.4)	84 (16.1)	27 (5.2)	
	Yes	424 (44.9)	239 (56.4)	69 (16.3)	78 (18.4)	38 (9.0)	0.056
Dur. Of	< 3	250 (26.5)	146 (58.4)	43 (17.2)	41 (16.4)	20 (8.0)	
Symp.	> = 3 & < 6	372 (39.4)	216 (58.1)	60 (16.1)	66 (17.7)	30 (8.1)	
(months)	> = 6	323 (34.1)	207 (64.1)	46 (14.2)	55 (17.0)	15 (4.6)	0.448
Leukoplakia	No	862 (91.2)	513 (59.5)	140 (16.2)	151 (17.5)	58 (6.7)	
	Yes	83 (8.8)	56 (67.5)	9 (10.8)	11 (13.3)	7 (8.4)	0.345
Smoking	No	592 (62.6)	342 (57.8)	97 (16.4)	106 (17.9)	47 (7.9)	
	Yes	353 (37.4)	227 (64.3)	52 (14.7)	56 (15.9)	18 (5.1)	0.166
SMF	No	904 (95.7)	538 (59.5)	142 (15.7)	160 (17.7)	64 (7.1)	
	Yes	41 (4.3)	31 (75.6)	7 (17.1)	2 (4.9)	1 (2.4)	0.081
Dur. Risk	0	162 (17.2)	93 (57.4)	24 (14.8)	27 (16.7)	18 (11.1)	
(months)	≤60	92 (9.7)	52 (56.5)	11 (12.0)	23 (25.0)	6 (6.5)	
	> 60 & ≤120	240 (25.4)	136 (56.7)	49 (20.4)	40 (16.7)	15 (6.3)	
	> 120 & ≤240	262 (27.7)	167 (63.7)	42 (16.0)	41 (15.7)	12 (4.6)	
	> 240	189 (20.0)	121 (64.0)	23 (12.2)	31 (16.4)	14 (7.4)	0.117
cTumor	UIG	386 (40.8)	221 (57.2)	69 (17.9)	70 (18.1)	26 (6.7)	
Growth	UPG	539 (57.0)	335 (62.1)	78 (14.5)	89 (16.5)	37 (6.9)	
	Other	20 (2.2)	13 (65.0)	2 (10.0)	3 (15.0)	2 (10.0)	0.710
c T Stage	T1	80 (8.5)	51 (63.7)	13 (16.3)	9 (11.2)	7 (8.8)	
	T2	252 (26.7)	145 (57.5)	52 (20.6)	46 (18.3)	9 (3.6)	
	T3	99 (10.5)	61 (61.6)	11 (11.1)	17 (17.2)	10 (10.1)	
	T4	514 (54.3)	312 (60.7)	73 (14.2)	90 (17.5)	39 (7.6)	0.102
Trismus	No	724 (76.6)	437 (60.4)	107 (14.8)	126 (17.4)	54 (7.5)	
	Yes	221 (23.4)	132 (59.7)	42 (19.0)	36 (16.3)	11 (5.0)	0.312
cSkinInv	No	721 (76.3)	429 (59.5)	121 (16.8)	125 (17.3)	46 (6.4)	
	Yes	224 (23.7)	140 (62.5)	28 (12.5)	37 (16.5)	19 (8.5)	0.333
OCF	No	916 (96.9)	549 (59.9)	145 (15.8)	159 (17.4)	63 (6.9)	
	Yes	29 (3.1)	20 (69.0)	4 (13.8)	3 (10.3)	2 (6.9)	0.738
cBoneInv	No	694 (73.4)	426 (61.4)	112 (16.1)	111 (16.0)	45 (6.5)	
	Yes	251 (26.6)	143 (57.0)	37 (14.7)	51 (20.3)	20 (8.0)	0.331
cNeck Node	No	233 (24.7)	171 (73.4)	33 (14.2)	23 (9.9)	6 (2.6)	
	Yes	712 (75.3)	398 (55.9)	116 (16.3)	139 (19.5)	59 (8.3)	< 0.001
Oral Site	BM	272 (28.7)	175 (64.3)	42 (15.4)	41 (15.1)	14 (5.2)	
	Tongue	200 (21.3)	110 (55.0)	33 (16.5)	38 (19.0)	19 (9.5)	
	Alvelobuccal	177 (18.7)	100 (56.5)	30 (17.0)	31 (17.5)	16 (9.0)	
	Alveolus	104 (11.0)	59 (56.7)	20 (19.2)	18 (17.3)	7 (6.7)	
	CA & FOM	90 (9.5)	62 (68.9)	9 (10.0)	14 (15.6)	5 (5.6)	
	RMT	54 (5.7)	37 (68.5)	7 (13.0)	8 (14.8)	2 (3.7)	
	Lip	48 (5.1)	26 (54.2)	8 (16.7)	12 (25.0)	2 (4.2)	0.551
Deg. of Diff.	WD	658 (69.6)	410 (62.3)	102 (15.5)	104 (15.8)	42 (6.4)	
	Others	287 (30.4)	159 (55.4)	47 (16.4)	58 (20.2)	23 (8.0)	0.194
Tumor Size	<=2	256 (27.1)	165 (64.4)	39 (15.2)	41 (16.0)	11 (4.3)	
(cm)	> 2 and < =4	466 (49.3)	270 (57.9)	86 (18.5)	80 (17.2)	30 (6.4)	
	> 4	223 (23.6)	134 (60.1)	24 (10.8)	41 (18.4)	24 (10.8)	0.022
Total		945 (100)	569 (60.2)	149 (15.8)	162 (17.1)	65 (6.9)	

*LC* Lower class, *LMC* Lower middle class, *UC* Upper class, *UMC* Upper middle class, *UIG* Ulceroinfiltrative, *UPG*, ulceroproliferative, *WD* Well Differentiated; *Deg. of Diff*. Degree of differentiation, *cSkin Inv* clinical skin involvement, *cBone Inv* clinical bone involvement, *SMF* sub mucous fibrosis.

Under multivariable regression analysis, proportional odds assumption was found to be satisfactory for each of the considered covariates selected for developing final model except histopathological tumor size. Moreover, overall proportionality assumption was not violated (*p* = 0.97). Accordingly, both the models (POM and PPOM) were developed and compared.

Interestingly, the results under proportional odds multivariable regression analysis emerged to be similar to those reported under binary form of nodal involvement [30]. To be more specific, pain at presentation, SMF, palpable neck node, oral site and degree of differentiation were retained to be significantly associated factors also with higher extent of nodal involvement. The patients presenting with pain were 37% more likely to have higher order of nodal positivity as compared to lower [1.37 (1.05 to 1.78)], whereas SMF was protective and patients with SMF had 57% less chance for higher frequency of node involvement [0.43 (0.21 to 0.90)]. Patients other than well differentiated tumors were more likely for presence of positive node [1.41 (1.07 to 1.86)] (Table 2).

**Table 2:** Factors associated with cervical lymph node involvement using proportional odds model

Variables		UOR(95%CI)	AOR(95%CI)*
Pain	No	1	1
	Yes	1.37 (1.07 to 1.77)	1.37(1.05 to 1.78)
SMF	No	1	1
	Yes	0.44 (0.22 to 0.90)	0.43 (0.21 to 0.90)
cNeck Node	No	1	1
	Yes	2.28 (1.66 to 3.14)	2.42 (1.73 to 3.39)
Oral Site	BM	1	1
	Tongue	1.53 (1.07 to 2.18)	1.83 (1.25 to 2.68)
	Alvelobuccal	1.42 (0.98 to 2.06)	1.19 (0.80 to 1.75)
	Alveolus	1.34 (0.86 to 2.07)	1.13 (0.72 to 1.80)
	CA & FOM	0.87 (0.52 to 1.43)	0.65 (0.38 to 1.11)
	RMT	0.84 (0.46 to 1.55)	0.78 (0.41 to 1.46)
	Lip	1.49 (0.83 to 2.67)	1.72 (0.94 to 3.13)
Deg. of Diff.	WD	1	1
	Others	1.34 (1.02 to 1.75)	1.41 (1.07 to 1.86)
Tumor Size	<=2	1	1
(cm)	> 2 and < =4	1.29 (0.95 to 1.75)	1.18 (0.86 to 1.63)
	> 4	1.36 (0.95 to 1.96)	1.18 (0.80 to 1.74)

*UOR* Unadjusted odds ratio, *AOR* Adjusted odds ratio.

*Adjusted in relation to smoking and clinical bone involvement

Like proportional odds model, under multivariable partial proportional odds model, pain at the time of presentation, SMF, palpable neck node, oral site and degree of differentiation were found to be significantly associated factors. In addition, tumor size also emerged to be significant. For patients with large tumor size, the chance of involving higher number of positive nodes went up. The patients with tumor size more than 4 cm had more than two fold chance to be involved with more than 4 numbers of positive nodes. The detailed results under multivariable regression analysis are presented in (Table 3).

**Table 3:** Factors associated with cervical lymph node involvement using partial proportional odds model

Variables		UOR(95%CI)	AOR(95%CI)*
Pain	No	1	1
	Yes	1.37 (1.07 to 1.77)	1.39(1.06 to 1.81)
SMF	No	1	1
	Yes	0.44 (0.22 to 0.90)	0.43 (0.21 to 0.90)
cNeck Node	No	1	1
	Yes	2.28 (1.66 to 3.14)	2.44 (1.75 to 3.42)
Oral Site	BM	1	1
	Tongue	1.53 (1.07 to 2.18)	1.83 (1.25 to 2.69)
	Alvelobuccal	1.42 (0.98 to 2.06)	1.19 (0.80 to 1.76)
	Alveolus	1.34 (0.86 to 2.07)	1.15 (0.73 to 1.83)
	CA & FOM	0.87 (0.52 to 1.43)	0.65 (0.38 to 1.12)
	RMT	0.84 (0.46 to 1.55)	0.77 (0.40 to 1.45)
	Lip	1.49 (0.83 to 2.67)	1.72 (0.95 to 3.13)
Deg. of Diff.	WD	1	1
	Others	1.34 (1.02 to 1.75)	1.42 (1.07 to 1.87)
Tumor Size	<=2	1	1
(cm)	> 2 and < =4	1.30 (0.96 to 1.77)	1.19 (0.87 to 1.64)
	> 4	1.20 (0.83 to 1.73)	1.01 (0.68 to 1.50)
	Cut2	1.69 (1.14 to 2.51)	1.47 (0.97 to 2.24)
	Cut3	2.39 (1.35 to 4.22)	2.12 (1.18 to 3.79)

*UOR* Unadjusted odds ratio, *AOR* Adjusted odds ratio. Cut2, category 4,3 vs. 2,1; Cut2, category 4 vs. 3, 2,1.; *Adjusted in relation to smoking and clinical bone involvement.

The comparative appraisal of both the models (Table 2, Table 3) revealed that partial proportional odds model stands to be preferred as indicated by LR and AIC (Table 4). Likelihood-ratio test also suggests the same (*p* = 0.004).

**Table 4:** Comparison of the developed models

Model	LR	AIC	BIC
POM	− 988.79	2011.57	2094.04
PPOM	− 983.18	2004.37	2096.54

### Assessment of the model

Fagerland and Hosmer test [34] for goodness of fit suggests (*p* = 0.88) that our developed model describes the distribution of nodal involvement satisfactorily. Further, link test suggest (*p* = 0.65) that there was no specification error in the developed partial proportional odds model. For discrimination ability of the developed model, Average Dichotomous C-index, Generalized C-index and Set wise C-index (ORC) were used [33]. The Average Dichotomous C-index values of the developed model was found to be 0.67, which suggests that probability to correctly discriminate a case with a lower outcome level from a case with higher outcome level was 0.67. However, Generalized C-index value was observed as 0.635, which means that probability to discriminate two cases from different categories was 0.635. Further, set wise C-index (ORC) value of the model was 0.634. It implies that the probability of correct discrimination in a pair of cases from two randomly chosen categories was 0.634.

### Validation of the developed model

As described earlier, temporal validation [43] of the developed model was evaluated on another dataset of 204 patients (validation data), collected prospectively from the same centre. The goodness of fit test on validation data also suggests (*p* = 0.35) that the developed model sustains its ability to describe the distribution of nodal involvement satisfactorily. In this case, the probability values of all the three indices (i.e. Average Dichotomous C-index, Generalized C-index and Set wise C-index (ORC)) providing discrimination ability of the ordinal logistic regression were 0.66, 0.63 and 0.64 respectively, which again indicates that the developed model performs equally on validation data. The analytical results amply reveal that the developed model remains to be generalizable and acceptable.

## Discussion

In biomedical research, in addition to frequent emergence of ordinal categorical data, sometime ordinal categories are the result of grouping of quantitative data [44]. However, as known in case of change in origin and scale, dichotomization or ignoring the order has its own disadvantage. Armstrong and Sloan (1989) found that compared to a cumulative logit model of a five level ordered response, logistic model attains only 50–75% of efficiency [29].

In this study, ordinal categories mainly relied on desired varying management strategies being adopted by the oncologists according to cut-off values. Accordingly, number of involved cervical nodes were categorized as 0, 1, 2–4 and > 4 involved nodes. Until now, there is no study considering the ordinal form of nodal involvement while assessing its associated factors. The available studies have dealt with only binary form of nodal involvement [2, 14, 24, 30, 45–47].

In the present study, POM seems to be appropriate as overall model did not violate the proportional odds assumption significantly. However, one of the covariates was found to violate this assumption. Theoretical ground or empirical tests do not provide clear guidelines about when to relax the proportional odds assumption [48]. Under exploration of this possibility, either to use POM or PPOM, log likelihood, AIC and likelihood ratio test supported the PPOM. Also, a clinically relevant covariate, tumor size emerged to be significant under PPOM. The availability of GOLOGIT2 with AUTOFIT syntax in STATA makes the things easier to select the appropriate model between POM and PPOM [35, 42].

Results under binary form of nodal involvement on the same dataset and considering same set of covariates are already reported elsewhere [30, 31]. Under appropriate consideration of the nodal involvement in the ordinal form, one of the clinically more relevant covariates i.e., histopathological tumor size, was also found to be significantly associated with extent of nodal involvement, which was missed in its binary consideration. However, other significant covariates were similar and also the effect size was in same direction. Under model assessment, model for binary as well as ordinal form of nodal involvement described the distribution satisfactorily.

## Conclusions

The analytical results under the present study reveal that in case of ordinal form of the outcome, ordinal regression may be a preferred choice. Further, in case of violation of proportionality assumption in any of the covariates, PPOM may be a better choice. This is likely to ensure accuracy not only in results but also in related inferences and their implications. In summary, pain at the time of presentation, sub mucous fibrosis, clinically palpable neck node, oral site, degree of differentiation and tumor size are the most probable associated factors with extent of nodal involvement in oral squamous cell carcinoma patients.

As mentioned earlier, the ordinal categories mainly relied on desired varying management strategies being adopted by the oncologists according to cut-off values of involved nodes. Otherwise, as true in case of any such study, change in cut-off values of nodal involvement is bound to change the results. Further, as true under such modeling, finite sample bias may remain a concern in these models as well.

## Data Availability

All the data are from the Department of Surgical Oncology, Dr. BRA-IRCH, All India Institute of Medical Sciences, New Delhi, India. The datasets are available upon request from SVS Deo.

## Abbreviations

POM: Proportional odds model
PPOM: Partial proportional odds model OSCC: Oral squamous cell carcinoma
RMT: Retromolar triangle
SMF: Sub mucous fibrosis
95%CI: 95% Confidence interval
LC: Lower class
LMC: Lower middle class
UC: Upper class
UMC: Upper middle class
UIG: Ulceroinfiltrative
UPG: Ulceroproliferative
WD: Well Differentiated
Deg. of Diff: Degree of differentiation
cSkin Inv: Clinical skin involvement
cBone Inv: Clinical bone involvement
UOR: Unadjusted odds ratio
AOR: Adjusted odds ratio
